# Analysis of the Correlation between Frontal Alpha Asymmetry of Electroencephalography and Short-Term Subjective Well-Being Changes

**DOI:** 10.3390/s23157006

**Published:** 2023-08-07

**Authors:** Betty Wutzl, Kenji Leibnitz, Daichi Kominami, Yuichi Ohsita, Michiko Kaihotsu, Masayuki Murata

**Affiliations:** 1 Graduate School of Information Science and Technology, Osaka University, Suita 565-0871, Japan; 2 Center for Information and Neural Networks (CiNet), National Institute of Information and Communications Technology, Suita 565-0871, Japan; 3 Institute for Open and Transdisciplinary Research Initiatives, Osaka University, Suita 565-0871, Japan; 4 Technology Innovation Center, Daikin Industries, Ltd., Settsu 566-8585, Japan

**Keywords:** subjective well-being, SWB, frontal alpha asymmetry, FAA, electroencephalography, EEG, environmental conditions

## Abstract

Subjective well-being (SWB) describes how well people experience and evaluate their current condition. Previous studies with electroencephalography (EEG) have shown that SWB can be related to frontal alpha asymmetry (FAA). While those studies only considered a single SWB score for each experimental session, our goal is to investigate such a correlation for individuals with a possibly different SWB every 60 or 30 s. Therefore, we conducted two experiments with 30 participants each. We used different temperature and humidity settings and asked the participants to periodically rate their SWB. We computed the FAA from EEG over different time intervals and associated the given SWB, leading to pairs of (FAA, SWB) values. After correcting the imbalance in the data with the Synthetic Minority Over-sampling Technique (SMOTE), we performed a linear regression and found a positive linear correlation between FAA and SWB. We also studied the best time interval sizes for determining FAA around each SWB score. We found that using an interval of 10 s before recording the SWB score yields the best results.

## 1. Introduction

Subjective well-being (SWB) is a subjective condition which everyone perceives differently and describes how people experience their lives in terms of how they think and feel. Diener et al. described SWB as “a broad category of phenomena that includes people’s emotional responses, domain satisfactions, and global judgements of life satisfaction” [1], p. 277. In the past, it was also known under different terms, such as “psychological well-being” [2], “quality of life” [3], or “subjective happiness” [4]. There are also discussions about differences and similarities with other concepts such as “comfort” or “quality of life”. Pinto et al. [5] summarize this discussion and point out that those terms often refer to each other in their definitions, e.g., the International Council of Nurses defines comfort as “sensation of physical ease and bodily well-being” [6]. Here, we stick to the term “subjective well-being”. In recent years, SWB has received more attention as it seems to affect many areas of our daily life. A worldwide analysis [7] showed that a lot of factors are included in SWB, such as demographic, institutional, personality, contextual, situational, environmental, and economic factors. There are also many papers focusing on specific aspects, e.g., psychology and public health [8]. The study of SWB has led to a deeper understanding and to a further subcategorization of SWB, namely into “evaluative well-being”, “experienced well-being”, and “eudaimonic well-being” as, for example, described in [9]. The assessment of SWB is usually done via a questionnaire amongst which the five-item World Health Organization Well-Being Index (WHO-5) is very popular, see [10] for a systematic review. In addition, as more attention is paid to SWB, a lot of research has been conducted focusing on psychological interventions to improve SWB, e.g., [11,12].

It is already known that SWB is related to biological phenomena. Some studies focused on metrics extracted from positron emission tomography (PET) [13] or functional magnetic resonance imaging (fMRI) [14], while others investigated a correlation between SWB and rapid eye movement (REM) sleep, salivary cortisol, and pro-inflammatory cytokines [15] or the relationship of SWB and neuroendocrine, inflammatory, cardiovascular activity, cortisol, or plasma fibrinogen [16]. Electroencephalography (EEG) seems to be a promising tool for measuring SWB, especially when focusing on the asymmetry of the frontal alpha waves. Tomarken et al. [17] measured the EEG of their participants twice, 3 weeks apart. They found that the group of participants who exhibited extreme relative left anterior activity compared to the right also reported an increased generalized positive affect. Wheeler et al. [18] focused on emotional reactivity and concluded that women with higher frontal activity in the left hemisphere reacted stronger when watching film clips designed to elicit positive emotions. A review by Davidson et al. [19] showed that the prefrontal cortex is asymmetrically involved when it comes to experiencing affect. Reznik and Allen [20] discuss frontal alpha asymmetry (FAA) as a predictor, outcome, mediator, or moderator for neural and psychological models as well as psychopathology. It was shown that FAA has a direct correlation to SWB [21,22]. The first of those two studies analyzed data from 84 right-handed adults and found that higher activity in the left superior frontal regions compared with the right side was associated with greater self-reported SWB [21]. The second study analyzes the effect of a positive psychological intervention. The authors measured SWB, depression, and anxiety at the beginning of the trial and again after 10 weeks. They found that improvement in SWB and relief of depression and anxiety were associated with an increase in FAA [22]. Focusing on other neuroimaging techniques, the fMRI study of Levesque et al. [14] and the PET study of Lane et al. [13] both also reported that the medial prefrontal cortex plays a crucial role for SWB.

In these studies, either different participants with individual SWB levels are studied, or interventions are conducted that last at least a few weeks when the studies address changes in an individual participant’s SWB. In this work, we do not aim for psychological interventions but rather investigate the effect of simple changes in the physical environment. This means, we assume that the psychological states of the participants stay the same and that the diverse environmental conditions changed during the experiment are the only sources for variation in SWB. Moreover, we decided to focus on EEG because it is an easy-to-use and inexpensive measurement technique that can be applied in realistic indoor environments.

There are studies that discuss the influence of specific EEG sensor locations, e.g., [23], and some studies even report findings for either depression or well-being in EEG frequency bands other than alpha, or in sensor locations other than the frontal areas [24,25]. However, we decided to focus on FAA in this paper because it is the most reported measure in the literature when it comes to finding a correlation between EEG and SWB. We investigate whether the reported relationship between FAA and SWB also holds when SWB is not changed by psychological interventions over several weeks, but rather changes in the physical environment on a minute or second scale. Moreover, we vary the time interval used for the FAA calculation to find out which intervals give the best results. We try to find out whether FAA only reflects an individual’s long-term psychological or psychiatric state or whether it also mirrors subtle short-term changes, such as feeling comfortable in a physical environment which might vary on a second or minute scale.

In summary, we aim to answer the following two main research questions, (RQ1) and (RQ2), in this paper.

(RQ1) *Does FAA also reflect subtle short-term changes in SWB?*

As mentioned above, most of the literature focuses on the correlation between FAA and SWB when SWB is either seen as a static metric from each participant or when participants undergo psychological or psychiatric interventions which lead to differences in SWB scores over weeks, months, or even longer. We are not trying to improve our participants’ depression scores or other psychological measures. We assume that these conditions remain the same during our experiments and, thus, reflect a background state with a static, unchanged score. We focus on short-term, non-persistent changes in SWB and see if we can relate changes in FAA to these small SWB changes. We hypothesize that there will be a positive linear correlation between FAA values and the corresponding SWB scores and try to confirm or deny this in this paper.

(RQ2) *Is there an ideal length and position for the time interval used to calculate FAA?*

The time interval over which FAA is calculated can vary. We will analyze different interval lengths and positions to find the interval that best reflects SWB. In other words, over what interval does FAA show the best correlation with subtle short-term changes in SWB?

## 2. Materials and Methods

### 2.1. Experiments

We conducted two experiments for our study: Experiment 1 in 2020 and Experiment 2 in 2022. Both experiments were approved by the local ethics committee at Osaka University. Experiment 1 took place in a typical office at the university with dimensions of around 7 m × 3.30 m. The room contained a meeting table and desk with a chair at which our participants were sitting. The main idea for the experiment was to measure brain functions of the participants during different SWB states. We attempted to influence the SWB states by changing the room environment to all combinations of temperature (22 °C, 24 °C, and 26 °C) and humidity (40%, 60%, and 80%). However, after recording sessions with the first seven participants, we realized that the settings with 80% were hard to maintain, so we changed them to 75%. A tolerance of ±0.5 °C was used for the temperature and ±5% for the humidity. All the equipment we used in this experiment was off-the-shelf equipment. For changing the temperature and humidity we used two heaters, two humidifiers, and one dehumidifier. Temperature and humidity were measured with a combined temperature and humidity meter. We started each session by bringing the room to the highest of the three humidity settings as this was the most difficult to achieve and so preparations were started before the participant arrived. Then, the humidity was kept stable for three runs of different temperature settings of 22 °C, 24 °C, and 26 °C. After having recorded all temperature settings at the highest humidity, we lowered the humidity to 60% and kept it stable while again going through all the three temperatures, from the lowest to the highest. After these three runs were finished, we lowered the humidity to 40% and performed the last three runs, with increasing temperature setting.

In Experiment 1 we measured 30 students (29 right-handed, 1 left-handed, 16 males, 14 females, ages 23.4 ± 3.3 years) from Osaka University. After each participant arrived, we first explained the experimental procedure to them. Then, the participant signed the consent form and sat on the chair. We used an EEG headset (EPOC+, EMOTIV, San Francisco, CA, USA) for our recording. This device has 14 channels, namely AF3, F7, F3, FC5, T7, P7, O1, O2, P8, T8, FC6, F4, F8, and AF4, following the standard 10–20 system with common mode sense (CMS) and driven right leg (DRL) references at locations P3 and P4, respectively. The device uses sequential sampling with a single analog-to-digital converter with an internal sampling rate of 2048 Hertz (Hz), downsampled to either 128 or 256 Hz. In the beginning of our experiment, it was not clear to us which of the sampling rates would yield better results. Thus, we tried different downsampling rates in different sessions. We found later that recording at different rates, or downsampling all sessions to the lower sampling rate at the beginning of the analysis, did not substantially change the results. Hence, we do not distinguish between sessions having different sampling rates in the following. The EEG bandwidth is 0.2–45 Hz with a digital notch filter at 50 and 60 Hz.

During the experimental runs all participants were asked to perform the same reading task of a newspaper to prevent distractions by any external stimuli. While the participant was sitting on the chair, the environmental conditions were set to one of the specific temperature–humidity pairs mentioned above. Once these settings were reached, the EEG recorded the brain state for five minutes. In addition, the participants were asked to rate their SWB every 60 s during the five-minute intervals on a scale from 1 to 10, with 1 being the worst and 10 being the best SWB. In other studies, SWB is often determined via a questionnaire. However, using even a short questionnaire is not feasible for us because we want to measure a short-term SWB on a 60 s (or even shorter) scale. Hence, we use the simplest form of an expanded Likert scale question [26], namely a verbal ranking from 1 to 10. This approach was chosen because it is the fastest way to get a response from the participants and the Likert scale in various forms is well established.

We described SWB to the participants in either Japanese or English, depending on their preference, as follows. An SWB score of 10 corresponds to feeling as comfortable as possible with the physical environment (temperature and humidity) and generally as well as possible. SWB 1, on the other hand, refers to the state of least comfort and not feeling well to the point of wanting to leave the current situation immediately. The numbers between 10 and 1 represent the gradual change between these states. This rating system and the general procedure were explained to the participants before starting the measurement. During the EEG recording, the experimenter reminded the participants every 60 s by saying the word “number” to report their SWB, which was recorded by the experimenter.

**Figure 1:**
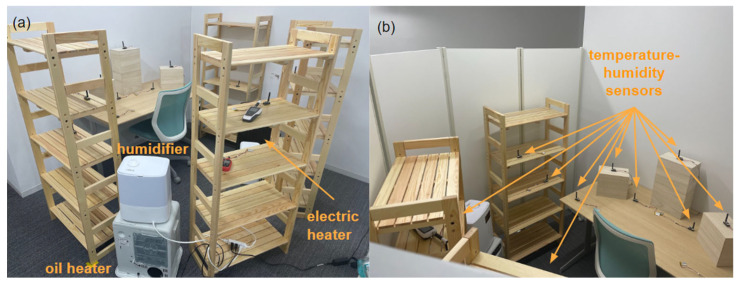
(**a**) Overview of the setup of Experiment 2 with heaters and humidifiers, and (**b**) the layout of the custom-made temperature-humidity sensors.

For Experiment 2 we made a few minor changes. We used a newer EEG headset, the EPOC X (EMOTIV, San Francisco, CA, USA), to measure the EEG signals. The EPOC X headset has the same technical features as the EPOC+. This time we decided at the beginning to record all sessions at a sampling frequency of 128 Hz. In addition, we used a different experimental room (Figure 1), which was divided by partitions into an area of 1.4 m × 2.1 m, with the idea that the temperature and humidity would be easier to control in a smaller space. Another change was the inclusion of custom-made temperature–humidity sensors [27]. The rest of the equipment (heaters and humidifiers) was the same as in the previous experiment.

We again recruited 30 students (28 right-handed, 2 left-handed, 16 males, 14 females, ages 22.3 ± 4.2 years) from Osaka University. None of those students participated in Experiment 1. The main procedure, i.e., the participant arriving, giving written consent to participate in the experiment, sitting on the chair, and performing a reading task, stayed the same. However, we slightly changed the temperature–humidity pairs from absolute to relative values. Hence, we measured combinations of three different temperatures (high, medium, low) and two different humidity levels (high, low), leading to a total of 6 combinations. See Table S1 in the Supplementary Materials for detailed information on the mean, standard deviation, and minimum and maximum temperature and humidity recorded during each run. We focused on changes in temperature and humidity rather than absolute values because we do not attempt to correlate absolute temperature or humidity values with any of the measured quantities, but rather use different temperature–humidity settings to provoke a change to the participants’ SWB. The frequency of collecting the SWB scores (again on a scale from 1 to 10) from the participants increased from 1 score every 60 s in Experiment 1 to 1 score every 30 s. The explanation of the experimental protocol and the SWB rating system were the same as in Experiment 1.

Table 1 below contrasts the two experiments in terms of participant characteristics and the tools used. Analysis 1, Analysis 2, and Analysis 3 will be described in Section 2.3.

### 2.2. EEG Processing

To preprocess our data, we wrote a MATLAB [28] pipeline making use of EEGLAB [29], HAPPE (The Harvard Automated Processing Pipeline for Electroencephalography) [30], and MARA (Multiple Artifact Rejection Algorithm) [31,32]. HAPPE is a standardized and automated pipeline for EEG recordings of variable lengths and artifact contamination levels, which is appropriate for us because we have short recordings from the participants and an EEG system that easily introduces artifacts. We refer the interested reader to [30] for a detailed explanation of the steps of HAPPE. At the end of our pipeline we turn to the computation of FAA by adapting the FAA EEGLAB toolbox by Michael Tesar [33], which automatically computes asymmetries of two channels from preprocessed EEG data following the description given in Section 2.5 in [34].

Our pipeline starts with loading the EEG data as EDF (European Data Format) files. Each EDF file consists of the data recorded by our EEG headset during one run, i.e., one temperature–humidity setting for one participant. If the sampling rate of the recording is 256 Hz, the first step is to downsample the data to 128 Hz. Then, all non-EEG channels are explicitly disabled, e.g., motion and other quality control measures that are also stored in the EDF file. The next step is to set the channel locations that will be needed later from the standard layout via sensor names. Then, the pipeline checks if the time series has a duration of at least 15 s to get rid of all runs that had to be stopped due to some problems. This is followed by detrending the data and then, as described in [30], we perform the first bad channel detection which makes use of the normed joint probability of the average logarithmic power (1–125 Hz). If a channel’s probability is more than 3 standard deviations away from the mean, it is considered a bad channel. Our pipeline also performs this bad channel detection twice to ensure that no bad channels are missed.

**Figure 2:**
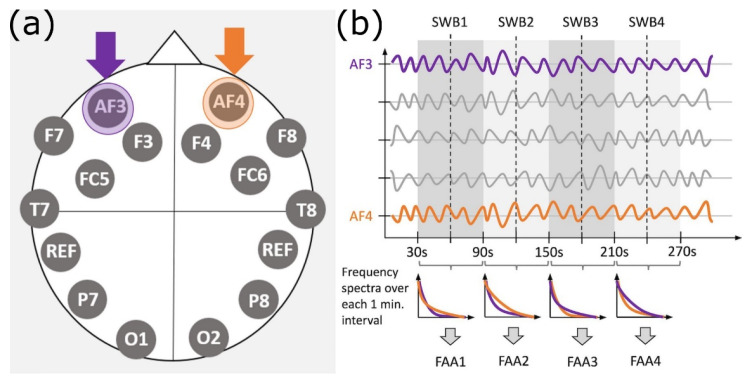
(**a**) EEG sensor layout. The frontal sensors AF3 (purple) and AF4 (orange) are the sensors used for the FAA calculation. (**b**) Overview of determining four pairs (FAA_i_, SWB_i_), i = 1, …, 4, from the pre-processed EEG channel data. The purple time series represents the AF3 channel and the orange time series stands for AF4. The time series shown in gray symbolize those of other unused channels. SWB scores were given by the participants every 60 s (Experiment 1), namely at 60 s, 120 s, 180 s, and so on (only the first 270 s are shown in the drawing). These time instants are indicated by the dashed vertical lines. The 60 s intervals were chosen so that the SWB scores were given in the middle of these intervals. The lower part of (**b**) shows the frequency spectra that result in FAA values corresponding to each SWB score.

Before continuing, the pipeline checks whether the channels AF3 and AF4, which are needed to compute FAA, are among the bad channels, in which case further processing is stopped (see Figure 2a for the EEG sensor layout). Then, a wavelet-ICA (independent component analysis) is performed. It removes all types of artifacts including signal discontinuities, high-amplitude artifacts such as eye-blinks, and any artifacts caused by the participant’s motion. This wavelet-ICA starts with an ICA and then performs a wavelet transform and thresholding before transforming back. The pipeline then uses MARA to automatically flag and reject independent components if the probability of having an artifact is larger than 0.50. This plugin is based on the idea that neural activity and artifactual components are generated independently and, thus, it carries out an automatic independent component classification by making use of a linear pre-trained classifier [31,32].

After these preprocessing steps, the pipeline selects time segments, e.g., for 60 s long intervals, interval 1 from 30 to 90 s of the EEG time series, interval 2 from 90 to 150 s, interval 3 from 150 to 210 s, interval 4 from 210 to 270 s, and so on. Then, the frontal channels AF3 and AF4 are selected. Following [33,34] a Fast Fourier Transform with a Hanning window of 50%, overlap is applied and the mean power density within the alpha band (8–13 Hz) is computed for both channels and for each interval. FAA is then calculated by subtracting the natural logarithm of the power density of the left side from that of the right side. Unlike [33], we decided not to use the absolute value of the difference of the log-transformed power spectra to also see the direction of the difference. Hence, the formula we used is

(1) $$FAA=mean\left(log\;pow_{R}-log\;pow_{L}\right)$$

with *log* standing for the natural logarithm, $pow_{R}$ being the power density of the signal on the right side, and $pow_{L}$ the power density of the left side. Figure 2b shows the selection of the channels and the time intervals, as well as the computation of the FAA values for the corresponding SWB scores.

### 2.3. Time Intervals for Determining FAA

In order to verify the stability of our results and also to see how using different time intervals for the FAA calculation will affect our results, we will perform the same analysis but calculate the FAA over different time intervals. In this section, we present three different analyses: Analysis 1, Analysis 2, and Analysis 3.

In Analysis 1, we use a 60 s interval for Experiment 1 and a 30 s interval for Experiment 2 around the given SWB (Table 2 and Table 3, second column). In Analysis 2, we investigate how different time intervals affect the results. We do not have a smaller time interval than 30 s for the SWB scores, but we shrink the interval over which we calculate the FAA, namely, we use intervals of lengths L = 5 s, 10 s, and 15 s. Moreover, we check two different approaches: (i) we calculate the FAA over the interval from L prior to the time t_SWB_ when the SWB score was recorded, i.e., [t_SWB_—L, t_SWB_], and (ii) we calculate the FAA of an interval over both sides of the time t_SWB_ when the SWB score was recorded, i.e., [t_SWB_—L/2, t_SWB_ + L/2]. In (i), the time of reporting the SWB score is at the end of the interval, see columns 3–6 in Table 3, and in (ii), the time of reporting the SWB score is in the middle of the interval, see columns 7–9 in Table 3.

**Figure 3:**
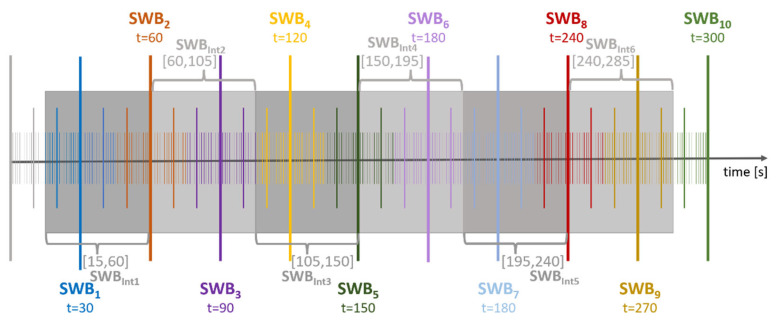
Illustration of choosing time intervals for Analysis 3. The time series continues until a maximum of 300 s and SWB is recorded every 30 s (Experiment 2). A different color represents a possibly different original SWB score SWB_k_ for k = 1, …, 10. The gray boxes show how the six 45 s intervals are aligned within the 300 s and the corresponding associated SWB_intk_ with k = 1, …, 6.

For Analysis 2 described above, we do not use the data of the entire EEG time series, but only data from some intervals. This results in omitting some data. It is not clear whether a larger interval would lead to better results. Therefore, we perform Analysis 3, in which we take the entire time series and divide it into intervals of length L = 5 s, 10 s, and k × 15 s, for k = 1, …, 20, i.e., we divide the time axis into as many intervals of length L as possible. Figure 3 shows an illustration of a full time series of 300 s and an interval length of 45 s when SWB scores were recorded every 30 s (Experiment 2). Considering a time series of 300 s, six full intervals of length 45 s can fit into it. We start at 15 s and add the six intervals continuously until we reach 300 s. What also needs to be considered in this approach is that different original SWB scores may fall within the same interval. We assume that the original SWB given at time t = 30 s is constant from t = 15 s until t = 45 s, after that we assume a different SWB from time t = 45 s until t = 75 s, and so on until the last original SWB. To associate an SWB_Int_ with the FAA from the given interval, we take the average over the original SWB scores given in that interval weighted by the number of seconds this SWB represents. This method results in having non-integer values for the SWB_Int_ which we then round to their nearest integer value, so that we can use the same analysis, as in Analysis 1 or 2.

### 2.4. Correlation between FAA and SWB

After determining the FAA values and their corresponding SWB scores, we now aim to find a relationship between those (FAA, SWB) pairs. We test our hypothesis that there will be a positive linear correlation between FAA values and the corresponding SWB scores (see RQ1). Looking at the dataset, we see that our data samples are not uniformly distributed. Typically, participants would report an SWB score of 6, 7, or 8 more often than 1, 2, 3, or 10. Therefore, running a linear regression on the given data would result in overfitting the central values and underfitting the others. Hence, we make use of an existing and well-established approach for overcoming such an imbalanced dataset, namely SMOTE (Synthetic Minority Over-Sampling Technique) [35]. We use the implementation in the imbalanced-learn [36] python [37] package, which is an open source library under the MIT license and relies on scikit-learn [38].

Before running SMOTE on each participant’s data individually, we make sure that each SWB score was given at least three times, so that we have at least three (FAA, SWB) pairs for each SWB reported by this participant. Otherwise, we remove all (FAA, SWB) pairs that contain this specific SWB score. This step is needed because SMOTE relies on a minimum number of nearest neighbors to produce new artificial data. We then check if there are at least three unique SWB scores among the given data in order to be able to perform a meaningful linear regression.

**Figure 4:**
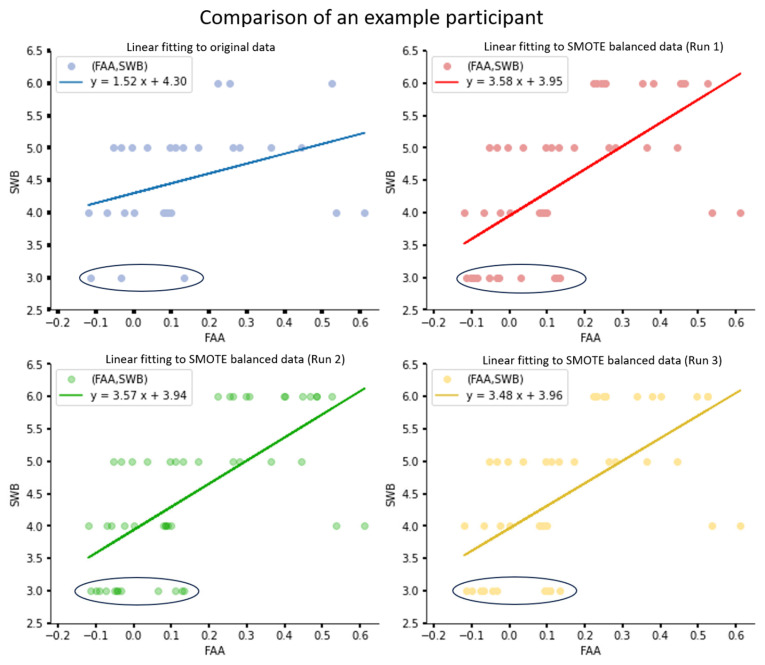
Comparison of the linear regression of Experiment 2 and Analysis 1. The dots in each subplot represent the (FAA, SWB) pairs of an example participant used for calculating the linear regression which is shown as a straight line. On the upper left-hand side, the linear regression of the original imbalanced data is given. The other three plots each show the result of linear regression after correcting for the imbalanced dataset using SMOTE. The number of (FAA, SWB) pairs in the original dataset are: #(FAA, 3) = 3, #(FAA, 4) = 11, #(FAA, 5) = 12, and #(FAA, 6) = 3 and after SMOTE #(FAA, SWB) = 12 for SWB of 3, 4, 5, and 6. The difference in the number of (FAA, SWB) pairs is highlighted for SWB = 3, see circled areas in all four subplots. When considering the original data, we can see that the linear regression favors values of SWB = 4 or 5. The results after SMOTE all show, while being numerically slightly different, a regression that does not favor the values in the center anymore and thus has a steeper line.

After these exclusion criteria, we apply SMOTE to the remaining data, resulting in the same number of (FAA, SWB) pairs per SWB score for each individual participant. Then, we perform a linear regression by making use of scikit-learn [38]. In Figure 4, we show a comparison of the linear regression of the data of an example participant with and without applying SMOTE. Without SMOTE, we can see that the middle values, in this case, SWB scores of 4 or 5, are favored, whereas, after applying SMOTE we do not see that phenomenon. Since the output of SMOTE is random, we run the same analysis 10 times per participant to get an average result. Running the same analysis one or even 100 times, does not change the results much, but it turns out that running the analysis 10 times is enough to get stable results. We end up with 10 slopes and intercepts per individual participant, which we then average for further processing. The statistical significance of the mean of these averaged slopes from all participants is tested with a one-sided *t*-test. The *p*-value and the 95% confidence interval (CI) are calculated using SciPy [39].

## 3. Results

Analysis 1 of Experiment 1 did not yield any statistically significant results. There is a tendency toward a positive linear correlation between FAA and SWB, but this correlation does not have a statistically significant *p*-value (*p* > 0.05). Hence in the following, we just focus on Experiment 2.

The first statistically significant result that we present is the analysis of the whole time series when FAA was calculated over 30 s intervals with SWB being in the middle of the time intervals (Analysis 1). Two participants were excluded because they were left-handed, and it is not clear how FAA measures of left-handed participants differ from measures from right-handed participants (see [25] for further information). A third participant was excluded because less than three different SWB scores were given. We got *p* = 0.016 with 26 degrees of freedom (df) and a CI of [0.26, Inf) with an average number of 66.37 (FAA, SWB) pairs per participant.

In the next parts, we present the results using different time intervals, i.e., Analysis 2 and 3 described in Section 2.3. In Analysis 2, intervals of length L = 5 s, 10 s, and 15 s were considered with the option that the SWB score was given at the end of the interval (marked as side = 1 in Table 3) or in the middle of the analyzed interval (marked as side = 2 in Table 3). The same three participants as before were excluded. Table 4 gives the *p*-value, df, and CI of each of the six analyses. One can see that if the interval of the FAA is taken before the SWB score is asked (side = 1 in Table 4), the *p*-value is lower, and the left endpoint of the CI is higher than for the side = 2 cases. All results are statistically significant (*p* < 0.05). The 10 s interval with side = 1 gives the best result with *p* = 0.0016, 26 df, a CI for the slope of the linear correlation of [0.52, Inf), and an average number of 66.48 (FAA, SWB) pairs per participant.

Analysis 3 uses the entire time series and also includes longer time intervals, see Section 2.3. Table 5 gives an overview of the determined *p*-values for the one-sided *t*-test, df, CI of the slope of the linear regression, and the average number of (FAA, SWB) pairs per participant for a chosen length of the intervals. The presented intervals of length L = 5 s, 10 s, and k × 15 s for k = 1 to 10 are shown in Table 5. The same three participants as before were excluded in all settings. However, the longer the interval length L, the more participants were excluded (see df in Table 5). For intervals longer than 150 s, analysis could not be performed because there were too few participants who would have met the inclusion criteria. In this analysis, we do not have a parameter “side” as in Analysis 2 because the time intervals can encompass more than one SWB score. Thus, it is not possible to center the interval around one value. The interval of 15 s gives the lower endpoint of the CI with the highest value and the interval of 10 s has a slightly lower *p*-value. These values are marked in bold in Table 5.

We also looked at the results of the two left-handed participants. The first participant had a negative slope of −0.91 in Analysis 1, whereas the same analysis for the second left-handed participant resulted in an average slope of 6.11 for the linear fit over all 10 SMOTE repetitions. A similar result was found for Analysis 2 and 3, namely, a slightly negative slope for the linear fit between FAA and SWB for the first of the two left-handed participants and a positive slope for the second left-handed participant.

## 4. Discussion

### 4.1. Experiment 1

In the analysis of Experiment 1, in which SWB was recorded every 60 s, we did not find any statistically significant results. Looking more closely at the data, we conclude that one reason for not finding statistical significance for this experiment could be the amount of available data. The number of available (FAA, SWB) pairs per participant for the analysis, when FAA was calculated over 60 s, was 36.26 with two participants having only 17 pairs. Since these numbers are very small, we expect that, if our hypothesis is correct, we will find statistically significant results when more data are available. Another reason for not finding a statistically significant correlation between FAA and SWB could be that there is no correlation and that our hypothesis is wrong. However, it may also be due to the time interval, i.e., a 60 s interval for the FAA calculation may be too long to correctly reflect subtle changes in SWB. These two possible reasons were improved in Experiment 2, in which the frequency of the recorded SWB scores was increased from 1 per 60 s to 1 per 30 s. This gives a higher temporal resolution of the SWB and thus might be more sensitive to capture slight changes in SWB, but it also increases the number of available (FAA, SWB) pairs per participant.

### 4.2. Experiment 2, Analysis 1

Focusing on Experiment 2, Analysis 1, where we calculated FAA over a 30 s interval with the SWB score recorded in the middle, showed a positive linear correlation between FAA and SWB with *p* = 0.016, df = 26, and CI = [0.26, Inf). This shows that the correlation between FAA and SWB also holds even when SWB is varied over environmental conditions and measured every 30 s, which means that we can answer our first research question (RQ1) from the Introduction: *Yes, FAA does reflect subtle short-term changes in SWB.* If we compare this result with the analysis of Experiment 1, we find that the average number of (FAA, SWB) pairs almost doubled from 36.26 to 66.37. This means that the lack of data could be the reason for not finding statistically significant results with the data of Experiment 1. However, we also increased the frequency of SWB which could also influence the result.

### 4.3. Experiment 2, Analysis 2

In the next part, we analyzed the influence of the chosen time interval on the results to find the best interval size and alignment. We tested several approaches. In Analysis 2, we focused on small interval lengths, namely 5 s, 10 s, and 15 s, and two different analysis methods, (i) with the SWB score reported at the end and (ii) in the middle of the analyzed time interval. All of those six different settings resulted in *p* < 0.05 and the lower bound for the CI was greater than 0. Going into more detail, we found that the worst result with the highest *p*-value and the lowest left endpoint of the CI was for the 5 s interval with the SWB score reported in the middle of the interval. The first reason for this is that it is a two-sided interval, which means that there are distortions in the time series due to the participants’ head movements when reporting their SWB. We preprocessed the raw data and tried to get rid of all artifacts, including movement artifacts, but it seems that we could not fully correct them, because all two-sided intervals give worse results than the one-sided intervals. Therefore, the artifacts, due to talking, affected the analyses, especially in the shortest time interval of 5 s. The other conclusion we can draw from this analysis is that a one-sided 10 s interval gives the best results, namely CI = [0.52, Inf) and *p* = 0.0016. Thus, it seems that a 10 s interval is ideal for our analysis in the absence of motion by the participant. Since all of these approaches yield statistically significant results, we can confirm the findings of Analysis 1 and also conclude that the relevance of this correlation can be improved by choosing shorter intervals before the time of reporting the SWB score.

### 4.4. Experiment 2, Analysis 3

In our final Analysis 3 we focused on time intervals of up to 300 s. Here, we assumed that the SWB score was stable for 30 s around the time when reported by the participant. We took the weighted average of all SWB scores in the chosen interval. The linear correlation between the FAA calculated over intervals of 5, 10, 15, 30, 45, 60, and 75 s and the corresponding averaged SWB scores all show a statistically significant positive linear correlation (*p* < 0.05, lower endpoint of CI > 0). The interval lengths of 60 and 75 s seem to mark a boundary between statistical significance and non-significance. Both of these intervals are still statistically significant, but their *p*-values are already close to 0.05 and the lower endpoint of the CI is close to 0. This is the same length of the time interval as in Experiment 1 with a similar number of (FAA, SWB) pairs per participant. Since the results were not statistically significant in Experiment 1 and are just below the cut-off in this analysis, we conclude that this interval length with the corresponding average number of (FAA, SWB) pairs is a boundary between statistical significance and non-significance.

Longer interval lengths, namely 90, 105, 120, 135, and 150 s, are all not statistically significant, and intervals longer than 150 s did not yield any results at all due to the exclusion criteria. There could be diverse reasons why the analysis of the interval length between 90 s and 150 s did not yield statistically significant results. The first reason is that such an interval is already too long to capture the small changes in brain functions that correlate with short-time SWB changes. However, we cannot fully confirm this because there are two other variables that contribute to having statistically significant results, and in our case, these are, first, the number of (FAA, SWB) pairs for each participant that go into the analysis, which probably also influenced the analysis of Experiment 1, and second, the df of the analysis. We can clearly see that the number of average (FAA, SWB) pairs per participant in the analysis decreases drastically as the interval length increases, starting with 419.22 for 5 s and ending with 13.25 for 150 s. Similarly, we can observe that the df decreases. For shorter interval lengths it remains quite stable with 26 for 5 s until 60 s and 24 for 75 s. Then it drops and so does the relevance of the results.

To see if these two factors are the main causes of the poor performance of the longer intervals, we limited the analysis of the shorter interval length to an input sample size of 20 (FAA, SWB) pairs and/or a df of eight by random sampling. In this case, even the analyses of the shorter intervals cannot maintain their statistically significant results. Thus, we cannot be sure which of the mentioned factors is the main reason for the lack of statistical significance. This means that intervals with a length of >75 s for the FAA calculation might already be too long to clearly represent the changes in SWB, but we cannot be sure because the small number of (FAA, SWB) pairs included in the analysis and the low df clearly influence the result. To further analyze this influence, we performed the same analysis with 100 randomly sampled (FAA, SWB) pairs per participant for interval lengths of 5 s, 10 s, and 15 s. When comparing the three results, there was no clear tendency for any of the three interval lengths to be superior to the other two. Therefore, the actual interval length over which we calculate the FAA does not seem to affect the result as long as the sample size and df are the same.

### 4.5. Comparing all Analyses Performed

When comparing the *p*-value and CI of all our analyses, it appears that the analysis of a 10 s interval before the time of the SWB recording yields the best results. We conclude that it is not the length of the interval that contributes to this result, but rather its location. Since the interval is located before the SWB score was reported, it eliminates all sorts of motion artifacts from saying the SWB score. We tried to get rid of such artifacts via preprocessing, but it seems that some are still present. In addition, the 10 s before the recorded SWB seems to be the most accurate time to be in a specific SWB state. If we select 10 s intervals continuously over the time series and associate an averaged SWB score with the computed *FAA* values, the result is still statistically significant, but has a higher *p*-value and a lower left endpoint of the CI. The reason may be that our assumption that SWB is stable for 30 s and then changes immediately is only an approximation. We can say that an analysis of about 70 (*FAA*, SWB) pairs with accurate SWB performs better than using 200 pairs with approximated SWB scores. This leads us to the answer of our second research question (RQ2) presented in the introduction: *The length for the time interval to compute the FAA seems to be less important than its temporal location. An interval over the 10 s prior to the SWB recording yielded the best results.* However, intervals of 5 s or 15 s prior to the SWB recording perform similarly well. When considering all possible 5 s, 10 s, or 15 s intervals in the time series, we get worse results than when just considering the ones before the SWB was recorded.

When it comes to left-handedness, we cannot draw any statistically significant conclusions because we only had two participants. We cannot even make an educated guess because the two participants we analyzed had contradictory results. This means that left-handed participants could also have a tendency towards positive linear correlation, and we just happened to measure a particular participant who does not show it. However, the opposite could also be true. A third possibility is that there is no tendency for either a positive or negative linear correlation between FAA and SWB when all left-handed participants are considered. 

All things taken together, we were able to answer both of our questions (RQ1) and (RQ2). We found that the correlation between FAA and SWB holds for small time intervals, with the specific interval location having the major influence on the performance of the model. Thus, we can say that the positive linear correlation between FAA and SWB holds not only on large time scales, when SWB is changed by psychological interventions over, e.g., weeks, as shown in the previous studies, but also when measured on small time scales, such as 30 s, when SWB is influenced by environmental conditions.

FAA is a relative measure. The higher FAA, the higher the activity in the left hemisphere also is. Affect is considered to be lateralized in the brain, with positive affect being predominantly in the left hemisphere and negative affect predominantly in the right hemisphere [40,41]. Greater activity in the left than in the right prefrontal cortex was found to be associated with dispositional positive affect [17]. Such an imbalanced activity is also related to a higher reaction to positively rated emotional stimuli [18,42]. Greater left than right prefrontal cortex activity also plays a role when it comes to suppressing or overcoming negative affect [43,44]. The first finding of our study, that FAA is high when SWB is also high, indicates higher left frontal activity when participants have higher SWB. Such an asymmetry was also reported by [21,22]. We would also like to point out that our study now indicates that this correlation also holds true when measuring short-term changes in SWB, namely on a 30 s scale, and when only the physical environment is changed. This means that the lateralization of brain activity adapts fast. This leads us to conclude that the correlation between FAA and SWB is not just a static phenomenon, but that it also follows slight short-term SWB changes in a given physical environment. This allows us to think about FAA not only as a measure in psychological or psychiatric studies, but also as a measure for everyday use that may one day find an application in smart homes, where *FAA* could be used to control the physical environment.

It should be noted that the results presented in this paper are group-level evaluations, which means that they do not automatically hold at the individual participant level. In fact, there were participants who did not show this specific correlation at the individual participant level. This may be partly due to the fact that SWB means something different to each individual. We have tried to be as clear as possible in our explanation to the participants, but we cannot be sure that we have completely eliminated these individual differences in interpretation in our participants. Especially in neuroscience, we see a high variability between individuals, e.g., in the localization of language processing areas or whenever the participant is left-handed [23]. The specific localization of the sensors used for the FAA computation also seems to be important, see [23] for a discussion. We decided to use an EEG headset in our study that was easy to use and quick to set up. This resulted in a trade-off between EEG preparation time and sensor density. Thus, an EEG headset with a higher sensor density might provide results with a higher accuracy. This also needs to be considered when thinking about a smart home application. The specific sensors and bands used in this study may not have been ideal choices for controlling the physical environment or may vary from person to person. However, our research shows that FAA calculated from the EEG sensors AF3 and AF4 could definitely be a candidate for such an application.

## 5. Conclusions

In this study we focused on short-term changes of SWB under different environmental conditions. We aimed to find a correlation between EEG recordings, more specifically the derived FAA, and SWB. Such a correlation has been described in the literature. However, we wanted to investigate whether such a correlation also holds for SWB in the sense of feeling comfortable and well in the physical environment, or if it can only be considered as a psychological or psychiatric measure. The results presented in this paper show that there is a positive linear correlation between FAA measured by EEG and short-term changes in SWB when reported every 30 s on a scale from 1 to 10 and varied by environmental conditions. This correlation was found to be strongest when FAA was calculated over a 10 s interval before the SWB was reported. This means that changes in FAA in correlation with SWB can also be seen on time scales of seconds. As mentioned in the previous sections, our results are at the group level and do not automatically apply to each individual participant. Thus, a future point of interest will be to discuss inter-subject variability as well as intra-subject variability. Furthermore, we plan to investigate different frequency bands in combination with all available EEG sensors to see if other combinations of frequency bands and EEG sensors might also correlate with short-term SWB changes.

## Figures and Tables

**Table 1:** Summary of the characteristics of the two experiments performed.

	Experiment 1	Experiment 2
Year performed	2020	2022
Number of total participants	30	30
Number of male participants	16	16
Number of female participants	14	14
Number of right-handed participants	29	28
Number of left-handed participants	1	2
Mean age (years)	23.4	22.3
Standard deviation of age (years)	3.3	4.2
Background of participants	University students	University students
SWB recording interval	60 s	30 s
EEG headset	EPOC+	EPOC X
Number of EEG sensors	14	14
EEG recording downsampling rate	128 or 256 Hz	128 Hz
Size of experimental room	7 m × 3.30 m	1.4 m × 2.1 m
Temperature and humidity measurement devices	1 combined T-H meter	15 custom-made T-H sensors
Maximum time per session (one participant)	4 h	4 h
Maximum time per run (T-H setting)	5 min	9 min
Different target T-H settings	9	6
Number of participants that completed all target T-H settings	21	28
Analysis 1 performed	Yes	Yes
Analysis 2 performed	No	Yes
Analysis 3 performed	No	Yes

**Table 2:** Overview of the time intervals used for determining the FAA for Analysis 1 and Experiment 1. The SWB score from 1 to 10 is denoted as SWB_i_ for i = 1, …, 4. Time intervals are given in seconds after the start of the EEG recording.

SWB	Interval
SWB_1_	[30, 90]
SWB_2_	[90, 150]
SWB_3_	[150, 210]
SWB_4_	[210, 270]

**Table 3:** Overview of the time intervals used for determining the FAA in Analysis 1 and 2 for Experiment 2. The SWB score from 1 to 10 is denoted as SWB_i_ for i = 1, 2, …. Time intervals are given in seconds after the start of the EEG recording. L denotes the length of the time interval. The parameter side = 1 if the time of the SWB recording was at the end of the interval and side = 2 stands for an interval where the SWB was recorded in the middle of the interval.

SWB	Interval						
	Analysis 1	Analysis 2					
		Side = 1 L = 5 s	side = 1 L = 10 s	Side = 1 L = 15 s	Side = 2 L = 5 s	Side = 2 L =10 s	Side = 2 L = 15 s
SWB_1_	[15, 45]	[25, 30]	[20, 30]	[15, 30]	[27.5, 32.5]	[25, 35]	[22.5, 37.5]
SWB_2_	[45, 75]	[55, 60]	[50, 60]	[45, 60]	[57.5, 62.5]	[55, 65]	[52.5, 67.5]
SWB_3_	[75, 105]	[85, 90]	[80, 90]	[75, 90]	[87.5, 92.5]	[85, 95]	[82.5, 97.5]
SWB_4_	[105, 135]	[115, 120]	[110, 120]	[105, 120]	[117.5, 122.5]	[115, 125]	[112.5, 127.5]
SWB_5_	[135, 165]	[145, 150]	[140, 150]	[135, 150]	[147.5, 152.5]	[145, 155]	[142.5, 157.5]
SWB_6_	[165, 195]	[175, 180]	[170, 180]	[165, 180]	[177.5, 182.5]	[175, 185]	[172.5, 187.5]
⁞	⁞	⁞	⁞	⁞	⁞	⁞	⁞

**Table 4:** Results of Analysis 2 of Experiment 2. The first column shows the chosen time interval length, i.e., L = 5 s, 10 s, or 15 s. The second column shows the sidedness of the interval, namely 1 if the SWB score was reported at the end of the chosen time interval or 2 if the SWB score was given in the middle of the time interval. The third column gives the corresponding *p*-value of a one-sided *t*-test, with df given in the fourth column. The next column gives the CI of the slope of the linear correlation. The hash symbol (#) in the last column stands for the average number of (FAA, SWB) pairs per participant. The analysis with the best fit (i.e., lowest *p*-value and highest value of the left endpoint of the CI) is given for a one-sided interval of 10 s and is marked in bold in the table.

L	Side	*p*-Value	df	CI	#
5	1	0.0017	26	[0.32, Inf)	72.55
**10**	**1**	**0.0016**	**26**	**[0.52, Inf)**	**72.55**
15	1	0.0056	26	[0.35, Inf)	72.55
5	2	0.0339	26	[0.05, Inf)	72.55
10	2	0.0198	26	[0.14, Inf)	68.48
15	2	0.0140	26	[0.23, Inf)	66.81

**Table 5:** Results of Analysis 3 of Experiment 2. The first and sixth columns show the selected interval length in seconds. The second and seventh columns show the corresponding *p*-values, followed by df and CI for the slope. The highest lower endpoint of the CI is given for a 15 s long interval and the lowest *p*-value for an interval length of 10 s, both are marked in bold in the table. The first seven interval lengths, i.e., 5, 10, 15, 30, 45, 60, and 75 s, all are statistically significant with *p* < 0.05. The hash symbol (#) in the last column stands for the average number of (FAA, SWB) pairs per participant.

L	*p*-Value	df	CI	#	L	*p*-Value	df	CI	#
5	0.0100	26	[0.192, Inf)	419.22	75	0.0332	24	[0.111, Inf)	26.44
**10**	**0.0083**	**26**	**[0.280, Inf)**	207.44	90	0.2188	23	[−0.444, Inf)	20.33
**15**	**0.0085**	**26**	**[0.311, Inf)**	138.96	105	0.2707	22	[−0.568, Inf)	18.87
30	0.0209	26	[0.200, Inf)	66.37	120	0.0612	6	[−0.112, Inf)	15.14
45	0.0134	26	[0.301, Inf)	45.48	135	0.4283	5	[−2.938, Inf)	13.33
60	0.0493	26	[0.004, Inf)	32.89	150	0.2183	4	[−1.033, Inf)	13.25

## Data Availability

The datasets generated during and/or analyzed during the current study are available from the corresponding author on reasonable request.

## Supplementary Materials

The following supporting information can be downloaded at: https://www.mdpi.com/article/10.3390/s23157006/s1. Table S1. In the first column we give the Part-Temp-Hum-Trial variable, where Part stands for the participant number, Temp for the temperature (L = low, M = medium, H = high), Hum for the humidity (L = low, H = high), and Trial for the trial number (some trials had to be repeated). The numbers in the first columns of the first seven rows are the intended absolute values. From row 8 on we started using relative values. The temperature and humidity recordings for 28-M-L-1 were accidentally deleted. This is marked as ‘-‘ in the table. T_mean_ stands for the mean temperature over the whole session and all used sensors. T_std_ gives the standard deviation and T_min_ and T_max_ the minimum and maximum value measured during the session. H_mean_ is analogously the mean humidity over the whole time of the session and all used sensors. H_std_ stands for the standard deviation and H_min_ respective H_max_ represent the minimum and maximum humidity values measured during this one session. T_mean_, T_std_, T_min_, and T_max_ are given in units of degree Celsius [°C] and H_mean_, H_std_, H_min_, and H_max_ in percent [%].
