# Supplementary material for: Analysis of the Correlation between Frontal Alpha Asymmetry of Electroencephalography and Short-Term Subjective Well-Being Changes
Source: Sensors (Basel). 2023 Aug 7;23(15):7006. doi: 10.3390/s23157006 (PMC10422288; doi:10.3390/s23157006)
Supplement: Supplementary file 1 [file sensors-23-07006-s001.zip › sensors-2468446-supplementary.pdf]

Supplementary Material

As mentioned in the main text, we used custom-made sensors developed by Peper et al. [27] for measuring temperature and humidity in Experiment 2. In Table S1 we give the mean values of the temperature and humidity of each session with the standard deviation, the maximum and minimum value recorded during each session.

Table S1. In the first column we give the Part-Temp-Hum-Trail variable, where Part stands for the participant number, Temp for the temperature (L = low, M = medium, H = high), Hum for the humidity (L = low, H = high) and Trial for the trial number (some trials had to be repeated). The numbers in the first columns of the first seven rows are the intended absolute values. From row 8 on we started using relative values. The temperature and humidity recordings for 28-M-L-1 were accidentally deleted. This is marked as '-' in the table. T_{mean} stands for the mean temperature over the whole session and all used sensors. T_{std} gives the standard deviation and T_{min} and T_{max} the minimum and maximum value measured during the session. H_{mean} is analogously the mean humidity over the whole time of the session and all used sensors. H_{std} stands for the standard deviation and H_{min} respective H_{max} represent the minimum and maximum humidity value measured during this one session. T_{mean}, T_{std}, T_{min}, and T_{max} are given in units of degree Celsius [°C] and H_{mean}, H_{std}, H_{min} and H_{max} in percent [%].

Part-Temp-Hum-Trial	T_{mean} [°C]	T_{std} [°C]	T_{min} [°C]	T_{max} [°C]	H_{mean} [%]	H_{std} [%]	H_{min} [%]	H_{max} [%]
01-20-60-1	20.23	0.96	18.02	22.35	54.81	3.56	48.34	67.11
01-20-50-1	21.87	0.93	20.04	23.39	51.01	3.34	45.87	61.10
01-20-55-1	20.00	0.89	18.24	22.28	60.37	3.89	50.56	72.59
01-20-60-1	19.47	0.87	17.67	21.10	63.13	3.34	55.16	70.65
01-19-60-1	19.12	0.93	17.20	21.04	64.07	3.77	56.04	71.00
01-23-55-1	23.56	1.11	21.16	25.28	52.74	3.97	45.38	63.93
01-26-50-1	24.96	1.25	22.05	27.27	51.65	4.18	45.02	64.63
01-L-L-1	20.71	0.51	19.27	21.88	25.23	0.93	23.30	27.67
01-M-L-1	23.50	0.56	22.01	24.33	26.03	1.01	24.12	28.38
01-H-L-1	25.99	0.97	24.32	28.34	25.01	1.11	22.57	26.79
02-M-H-1	23.42	0.69	22.05	24.99	63.16	8.44	46.04	75.90
02-L-H-1	21.45	0.50	20.44	22.45	62.19	4.06	52.76	72.89
02-L-L-1	20.62	0.45	19.32	21.33	32.04	1.50	28.72	35.99
02-M-L-1	23.58	0.62	22.15	24.59	32.64	1.13	29.78	35.10
03-L-H-1	20.83	0.64	18.99	21.90	52.53	5.11	41.98	67.06
03-M-H-1	22.99	0.71	21.34	24.82	53.25	5.96	40.03	83.69
03-H-H-1	25.62	0.75	24.30	27.46	46.43	3.51	38.80	55.09
03-L-L-1	20.67	0.60	19.35	21.96	25.00	0.93	22.68	27.15
03-M-L-1	23.46	0.71	21.66	24.37	26.11	1.15	23.92	28.91
03-H-L-1	25.70	0.78	23.78	26.87	25.77	0.85	24.65	27.75
04-L-H-1	20.77	0.45	19.84	21.55	57.72	3.29	48.79	65.33
04-M-H-1	23.62	1.01	22.01	25.14	52.37	6.07	42.37	63.61
04-M-H-2	23.55	1.13	21.63	25.51	54.21	7.35	41.09	68.82
04-H-H-1	26.20	1.33	23.66	28.72	48.55	7.08	36.50	65.95

04-M-L-1	22.58	0.43	21.54	23.62	21.85	0.98	18.81	23.91
04-H-L-1	26.38	0.95	24.79	29.10	23.19	1.00	20.80	25.59
05-L-H-1	19.50	0.50	18.18	20.30	55.96	3.91	48.17	67.41
05-M-H-1	23.30	0.89	21.85	24.80	51.23	5.46	38.26	67.72
05-H-H-1	25.96	0.86	24.09	27.51	46.64	3.81	40.74	54.68
05-L-L-1	21.09	0.46	19.72	21.79	27.66	0.78	26.04	30.05
05-M-L-1	23.53	0.83	21.75	25.18	27.42	1.32	24.34	30.40
05-H-L-1	25.97	0.66	24.72	27.11	26.89	0.80	25.40	28.63
06-L-H-1	19.42	0.49	18.19	20.30	58.45	2.91	52.41	67.94
06-M-H-1	23.43	0.92	21.89	25.23	56.04	3.81	46.44	63.19
06-H-H-1	26.17	0.90	24.30	27.93	51.90	3.13	44.85	60.25
06-L-L-1	20.61	0.54	19.65	21.73	38.80	1.10	35.77	40.89
06-L-L-2	20.50	0.51	19.35	21.56	38.70	1.12	36.12	41.42
06-H-L-1	26.11	0.85	24.16	28.03	34.71	1.34	32.08	38.00
06-M-L-1	22.42	0.55	20.81	23.76	34.31	0.93	32.35	37.65
07-L-H-1	21.09	0.47	20.11	21.90	72.10	3.09	63.29	79.43
07-M-H-1	23.64	0.82	22.24	25.06	67.79	5.72	56.48	80.27
07-H-H-1	25.71	1.14	23.27	27.59	63.69	7.69	51.21	79.26
07-L-L-1	20.74	0.41	19.60	21.45	38.64	0.71	37.54	40.74
07-M-L-1	22.90	0.53	21.72	23.86	39.66	1.10	36.71	41.64
07-M-L-2	23.54	0.61	22.20	24.95	38.49	1.48	34.91	40.76
07-H-L-1	26.15	0.67	24.95	27.25	37.62	1.19	35.26	40.27
08-L-H-1	20.67	0.59	19.27	21.56	68.55	3.60	57.81	77.44
08-M-H-1	23.04	0.95	21.49	24.95	64.40	6.65	49.97	75.72
08-H-H-1	26.00	1.34	23.77	28.63	59.67	8.03	43.25	74.97
08-L-L-1	20.99	0.45	20.00	21.76	31.49	0.68	30.29	33.05
08-M-L-1	23.57	0.74	22.01	25.04	32.35	1.52	28.89	34.65
08-H-L-1	25.94	1.04	24.20	27.97	30.47	1.90	26.59	34.26
09-L-H-1	19.52	0.54	18.57	20.48	62.72	6.64	53.12	75.64
09-M-H-1	22.68	1.10	21.07	24.77	60.78	7.82	46.61	75.67
09-H-H-1	25.48	1.26	23.21	27.98	56.63	8.85	42.19	71.39
09-L-L-1	21.43	0.56	20.07	22.31	28.70	1.00	26.75	30.51
09-M-L-1	23.53	0.51	22.15	24.35	29.68	0.75	27.66	31.38
09-H-L-1	26.15	0.65	25.05	27.45	28.13	1.09	25.71	30.37
10-L-H-1	21.07	0.48	19.64	21.90	63.07	4.30	47.73	73.07
10-M-H-1	22.83	0.71	21.61	24.24	59.49	7.11	45.02	72.84
10-M-H-2	22.87	0.71	21.78	24.11	57.51	6.68	43.39	72.14
10-H-H-1	25.31	1.18	23.16	27.54	55.19	8.95	41.80	71.08
10-L-L-1	20.98	0.44	19.74	21.89	23.81	0.79	22.00	25.55
10-M-L-1	23.35	0.58	21.89	24.48	25.90	0.82	23.98	27.75
10-H-L-1	26.11	1.01	23.64	28.34	25.16	1.50	22.04	28.42
11-L-H-1	19.44	0.61	18.13	20.63	56.61	4.43	44.63	66.35
11-L-H-2	19.44	0.49	18.28	20.25	56.40	3.91	46.75	63.70
11-M-H-1	22.78	1.19	20.57	25.61	55.30	9.21	39.15	71.21
11-H-H-1	25.26	1.40	22.50	28.86	50.71	9.80	33.14	67.85

11-L-L-1	20.25	0.86	17.98	21.83	26.43	1.06	24.75	29.16
11-M-L-1	23.44	0.68	22.08	24.85	27.38	1.36	24.34	29.52
11-H-L-1	26.24	0.79	24.91	28.01	25.65	1.48	22.39	28.60
12-L-H-1	19.91	1.59	14.32	22.04	55.79	6.00	40.74	71.70
12-M-H-1	22.96	0.94	21.34	24.85	52.85	8.17	39.72	66.97
12-H-H-1	25.38	0.99	23.57	27.92	47.49	7.23	35.12	60.43
12-L-L-1	20.85	0.58	19.27	22.35	24.86	0.84	23.21	26.79
12-M-L-1	23.46	0.55	21.96	24.81	26.26	1.05	23.80	28.56
12-H-L-1	25.79	0.79	24.29	27.54	25.20	1.42	22.39	28.42
13-L-H-1	18.79	0.39	17.95	19.54	50.81	3.34	44.28	59.50
13-M-H-1	23.01	0.89	21.61	24.92	51.00	6.51	37.03	63.09
13-H-H-1	25.47	0.96	23.73	27.63	48.81	6.34	35.97	61.49
13-L-L-1	20.58	0.48	19.32	21.93	26.31	0.89	23.92	28.56
13-M-L-1	23.52	0.79	22.05	25.18	27.76	1.40	24.69	30.91
13-H-L-1	26.18	0.71	24.96	27.60	26.67	1.39	23.41	28.96
14-L-H-1	20.72	0.49	19.51	21.47	55.51	3.90	47.28	64.36
14-M-H-1	23.19	0.77	21.91	25.04	52.69	4.38	41.98	60.44
14-H-H-1	25.89	1.08	24.15	28.34	49.12	6.30	37.20	60.43
14-L-L-1	21.52	0.43	20.31	22.50	24.17	0.75	22.53	26.08
14-M-L-1	23.90	0.64	22.34	25.37	26.36	1.03	23.80	28.86
14-H-L-1	26.02	0.76	24.84	27.59	25.77	1.27	22.92	28.25
15-L-H-1	19.80	0.45	18.94	20.58	48.74	3.75	40.03	57.78
15-M-H-1	23.07	0.92	21.59	25.33	47.02	6.01	35.97	59.37
15-H-H-1	25.01	0.85	23.08	27.02	45.43	6.40	33.67	57.78
15-L-L-1	20.80	0.45	19.51	21.70	25.80	0.71	24.45	27.32
15-M-L-1	23.89	0.80	22.69	26.03	25.95	1.37	22.74	28.33
15-H-L-1	25.83	0.76	24.59	27.97	24.67	1.21	21.86	27.54
15-H-L-2	24.93	0.52	23.84	26.13	24.68	0.72	23.06	26.13
16-L-H-1	21.54	0.49	20.64	22.37	56.12	4.91	41.72	64.36
16-H-M-1	24.01	0.87	22.53	25.56	51.66	7.02	39.01	63.43
16-H-H-1	25.81	0.98	23.73	27.65	49.22	8.09	35.44	63.08
16-L-L-1	20.39	0.90	17.50	21.92	28.86	1.34	26.22	33.16
16-M-L-1	23.51	0.56	21.68	24.43	27.04	0.85	25.18	29.62
16-H-L-1	26.02	0.57	24.84	26.88	25.71	0.92	23.63	27.89
17-L-H-1	22.17	0.72	21.07	23.60	50.61	5.16	40.74	60.39
17-M-H-1	24.85	0.92	23.16	27.37	53.33	6.96	37.91	67.15
17-H-H-1	27.24	1.00	25.38	29.68	50.33	7.14	35.26	62.90
17-L-L-1	22.32	0.49	20.69	23.20	26.34	0.82	25.00	28.56
17-M-L-1	25.35	0.67	23.85	26.69	27.28	1.21	24.69	29.44
17-H-L-1	27.90	0.74	26.34	29.43	25.74	1.27	23.06	28.42
18-L-H-1	22.74	0.49	21.68	23.45	50.36	3.24	42.42	58.79
18-M-H-1	25.33	1.08	23.66	27.87	48.36	6.61	35.97	60.16
18-H-H-1	27.25	0.99	25.36	29.10	47.07	6.67	35.08	57.51
18-L-L-1	21.54	0.87	18.55	23.35	27.95	1.21	25.59	32.35
18-M-L-1	25.50	0.61	23.99	26.71	25.88	1.07	23.59	28.03

18-H-L-1	27.89	0.67	26.59	29.10	24.51	1.16	22.04	26.83
19-L-H-1	20.88	0.46	19.65	21.62	52.83	3.40	44.63	62.68
19-M-H-1	24.20	0.84	22.93	25.89	52.39	5.37	40.43	60.07
19-M-H-2	24.00	0.79	22.59	25.47	53.45	7.14	40.21	66.44
19-H-H-1	26.50	0.67	25.33	28.06	48.84	6.62	35.79	62.46
19-L-L-1	20.72	0.98	17.28	22.78	30.93	1.65	27.81	37.38
19-M-L-1	24.71	0.65	23.19	26.65	29.02	1.25	25.75	31.21
19-H-L-1	26.81	0.75	25.42	29.43	27.88	1.44	24.16	31.08
20-L-H-1	22.09	0.48	20.88	22.79	60.22	3.50	51.53	68.78
20-M-H-1	24.50	0.79	23.00	26.36	56.17	5.95	41.62	66.62
20-H-H-1	26.84	0.92	25.05	28.91	54.00	6.74	39.54	66.35
20-L-L-1	22.20	0.58	20.88	23.15	29.81	1.08	27.99	31.38
20-M-L-1	25.04	0.74	23.47	27.12	29.22	1.43	25.71	31.38
20-H-L-1	26.92	1.02	25.64	29.71	28.41	1.70	23.98	30.90
20-H-L-2	26.78	0.81	25.55	28.72	27.92	1.45	24.83	30.55
20-H-L-3	26.26	0.49	25.40	27.40	27.79	0.97	25.53	29.84
20-H-L-4	27.14	0.57	26.06	28.58	27.71	1.20	24.83	30.02
21-L-L-1	22.74	0.46	21.82	23.31	46.36	0.87	45.01	48.27
21-M-L-1	24.41	0.76	23.00	26.08	45.07	1.89	40.07	49.15
21-H-L-1	26.84	1.06	24.93	29.24	42.08	2.36	36.18	47.34
21-L-H-1	22.72	0.52	21.35	23.63	65.26	3.31	53.47	74.48
21-M-H-1	24.29	0.62	22.76	25.48	63.53	5.20	52.41	75.01
21-H-H-1	26.97	0.95	25.47	29.02	60.22	5.49	47.28	71.74
21-H-H-2	26.95	0.73	25.73	28.69	60.94	5.41	48.17	70.86
21-H-H-3	26.88	0.58	25.50	27.89	62.22	4.86	50.82	71.83
22-L-H-1	22.69	0.49	21.58	23.63	71.74	2.77	65.87	79.21
22-M-H-1	24.56	0.83	23.04	26.22	68.60	5.22	54.53	78.86
22-H-H-1	26.93	1.13	25.12	29.71	66.65	7.28	48.97	79.96
22-L-L-1	20.85	1.19	17.22	22.92	48.25	2.40	43.72	57.28
22-M-L-1	24.91	0.69	23.37	26.41	44.34	1.70	39.72	46.77
22-H-L-1	27.30	0.87	25.88	29.05	42.23	2.19	37.24	46.11
23-L-H-1	22.73	0.53	21.25	23.53	74.04	2.66	66.47	81.51
23-M-H-1	24.59	0.65	22.95	25.56	69.33	4.29	59.31	79.92
23-H-H-1	26.89	0.76	25.59	28.20	67.52	5.10	56.16	77.76
23-L-L-1	21.73	1.31	16.99	23.77	49.84	3.56	44.25	65.49
23-M-L-1	25.26	0.53	23.56	26.08	45.52	1.37	42.19	48.97
23-H-L-1	27.12	0.59	25.73	28.48	44.23	1.62	39.54	47.34
24-L-H-1	22.64	0.48	21.68	23.44	72.75	2.98	65.34	78.55
24-M-H-1	24.70	0.76	23.19	25.94	71.52	5.40	57.18	82.09
24-H-H-1	26.88	1.02	25.17	28.77	69.15	6.66	54.53	80.94
24-L-L-1	22.47	0.63	20.81	23.86	42.85	1.17	40.49	45.78
24-M-L-1	25.14	0.57	23.47	26.41	41.23	1.32	37.60	43.59
24-H-L-1	27.26	0.81	25.99	28.96	39.43	1.74	34.94	42.92
24-H-L-2	27.45	0.73	26.08	29.24	38.47	1.76	34.24	42.39
25-L-L-1	22.39	0.45	21.53	23.08	34.38	0.66	33.47	36.64

25-M-L-1	24.40	0.73	23.09	25.56	33.83	1.35	30.70	36.64
25-H-L-1	26.94	0.95	25.19	28.58	31.76	1.72	28.40	35.67
25-L-H-1	21.32	0.61	19.84	22.49	60.46	5.12	46.07	72.46
25-M-H-1	24.43	0.69	23.00	25.80	56.30	6.54	41.66	68.92
25-H-H-1	26.77	1.04	25.02	29.43	55.26	6.93	38.09	68.25
26-L-L-1	21.84	0.60	20.42	23.01	39.81	1.22	37.36	42.83
26-M-L-1	24.60	0.83	22.95	26.50	37.13	1.75	32.96	40.71
26-H-L-1	27.15	1.21	25.45	29.80	34.81	2.28	29.78	38.59
26-L-H-1	21.91	0.43	20.77	22.87	58.43	3.75	45.89	70.62
26-M-H-1	23.31	0.71	21.86	24.76	65.60	4.32	55.46	76.25
26-H-H-1	26.79	0.90	24.64	28.81	62.08	5.77	45.38	74.84
27-L-L-1	22.94	0.43	22.02	23.50	33.87	0.64	32.88	35.41
27-M-L-1	24.77	0.62	23.37	25.84	33.34	1.05	30.52	35.89
27-H-L-1	27.25	0.85	25.69	29.14	31.64	1.40	28.05	34.44
27-L-H-1	21.16	0.42	20.21	22.06	58.37	4.25	44.12	73.81
27-L-H-2	21.72	0.50	20.44	22.72	61.46	4.12	50.20	75.37
27-M-H-1	24.49	0.74	23.37	25.94	58.82	5.75	43.08	69.53
27-H-H-1	26.71	0.97	24.73	28.72	56.47	6.22	41.49	69.53
28-L-L-1	22.15	0.50	20.92	22.87	29.98	0.67	28.89	31.92
28-M-L-1	-	-	-	-	-	-	-	-
28-H-L-1	27.28	1.44	25.41	30.84	29.39	2.17	23.80	32.14
28-H-L-2	27.28	1.08	25.55	30.18	28.87	1.73	24.34	31.96
28-L-H-1	21.60	0.47	20.26	22.87	57.63	4.50	43.95	74.16
28-M-H-1	24.41	0.63	22.90	25.70	56.95	5.96	40.43	70.06
28-H-H-1	27.18	1.02	25.16	29.24	54.29	6.21	39.15	66.30
29-L-L-1	21.80	0.42	20.97	22.37	33.84	0.79	32.94	36.11
29-L-L-2	21.97	0.38	21.02	22.46	33.95	0.73	33.16	35.94
29-M-L-1	24.57	0.73	23.23	26.03	33.44	1.34	29.99	35.98
29-H-L-1	27.26	0.90	25.57	29.43	31.94	1.52	27.69	34.79
29-L-H-1	21.13	0.40	20.07	22.13	58.67	4.39	46.42	72.89
29-M-H-1	24.14	0.78	22.00	25.99	56.31	6.04	39.9	70.24
29-H-H-1	26.81	1.17	24.55	29.66	54.30	7.21	37.91	68.60
30-L-L-2	21.30	0.77	19.89	22.68	31.93	1.41	28.76	34.44
30-M-L-1	24.08	1.01	22.17	26.22	30.27	1.77	26.10	34.08
30-H-L-1	26.90	1.12	24.67	29.33	28.13	1.82	24.16	32.31
30-L-H-1	20.25	0.89	18.42	21.47	63.85	7.42	44.19	77.84
30-M-H-1	23.89	1.06	21.81	26.41	56.25	7.99	37.60	72.89
30-H-H-1	26.61	1.23	23.93	29.33	53.43	8.08	36.67	69.00
